# Assessing the Activity of Antimicrobial Peptides Against Common Marine Bacteria Located in Rotifer (*Brachionus plicatilis*) Cultures

**DOI:** 10.1007/s12602-022-09928-2

**Published:** 2022-05-25

**Authors:** Chelsea Woods, Lindsey Woolley, Gavin Partridge, Mengqi Chen, Evan F. Haney, Robert E. W. Hancock, Nicky Buller, Andrew Currie

**Affiliations:** 1 College of Science, Murdoch University, Engineering & Education, HealthPerth, WA Australia; 2 Department of Primary Industries and Regional Development, Fremantle, Perth, WA 6160 Australia; 3 Centre for Microbial Diseases and Immunity Research, Department of Microbiology and Immunology, University of British Columbia, Vancouver, BC Canada; 4 Centre for Molecular Medicine and Innovative Therapeutics, Murdoch University, Perth, WA Australia; 5 Wesfarmers Centre for Vaccines and Infectious Diseases, Telethon Kids Institute, Perth, WA Australia

**Keywords:** Antimicrobial peptides, *Brachionus plicatilis*, Minimum inhibitory concentration, Antibacterial activity, Probiotics

## Abstract

Rotifers are used as the first feed for marine fish larvae and are grown in large cultures that have high loads of organic matter and heterotrophic bacteria; these bacteria are passed on to the developing fish larvae and can potentially lead to bacterial infections. A modified minimum inhibitory concentration (MIC) protocol for antimicrobial peptides was used to determine the potency of ten antimicrobial peptides (AMPs) in artificial seawater relevant to a rotifer culture (salinity of 25‰) against common marine pathogens. All of the AMPs had antimicrobial activity against the bacterial isolates when the salt concentration was approximately zero. However, in high salt concentrations, the majority of the AMPs had an MIC value greater than 65 µg mL^−1^ in artificial seawater (25‰). The only exceptions were 2009 (32.5 µg mL^−1^) and 3002 (32.5 µg mL^−1^) against *Vibrio rotiferianus* and *Tenacibaculum discolor*, respectively. The selected synthetic AMPs were not effective at reducing the bacterial load in brackish salt concentrations of a typical commercial rotifer culture (25‰).

**Supplementary Information:**

The online version contains supplementary material available at 10.1007/s12602-022-09928-2.

## Introduction

The aquaculture industry has increased dramatically over the decades due to the need to counter the effects of overfishing and respond to the growing demand for quality protein [1]. The rising demand for seafood has led to intensification of aquaculture farming practices, particularly for crustaceans and fish. Increased harvest capacity is often achieved by increasing stocking densities, yet this may lead to health implications and disease outbreaks. Such outbreaks result in large economic losses and therefore constrain industry growth [2–4]. Use of antibiotics and chemotherapies that were historically used to mitigate such disease outbreaks has rapidly declined in recent years and is now banned in many countries due to the development of antimicrobial resistance [5, 6]. This decreased use of antibiotics has coincided with increased research efforts to develop novel antimicrobial therapies including vaccines [7, 8], probiotics [9], medicinal plants [10] and antimicrobial compounds [5].

Antimicrobial peptides (AMPs) are an ancient part of the natural defence systems of most organisms and have a broad spectrum of antimicrobial properties [11–13]. These peptides often act through electrostatic forces and cause rapid microbial cell lysis, reducing the chance of bacteria acquiring resistance [14–16]. Additionally, AMPs degrade rapidly, unlike antibiotics that reside in the environment and bio-magnify throughout the food chain [5, 17]. The application of natural AMPs is limited because of their susceptibility to the cation inferences which occurs in solutions such as seawater, blood and serum [18, 19]. To overcome this, synthetic peptides have been developed with modifications to improve the overall characteristics of the peptide, including improved antimicrobial activity, reduced cytotoxicity or haemolysis, increased salt tolerance and enhanced cell selectivity [20–25]. The modification of AMPs to tolerate high salinities, such as those found in marine environments, would expand their potential application in aquaculture systems.

According to Zermeño-Cervantes et al. [22], 216 articles have been published on the use of AMPs in aquaculture. This research has concentrated on in vitro assessments of antibacterial activity of common marine pathogens [26–28], functionality of AMPs as part of the innate immune system in aquaculture species [29, 30] and their mechanisms of action [16, 31]. There are only a few in vivo co-culture studies, with most assessing the survival of the host after the administration of an AMP and a common pathogen, rather than the direct antimicrobial activity, per se [32, 33].

Hatchery production is a critical phase in aquaculture of marine fish as fish larvae are very underdeveloped, with a simple alimentary canal and a naïve and developing adaptive immune system. As such, larvae are highly susceptible to the bacterial communities in their environment (i.e. in the rearing water and in the feed they consume), and some of these bacterial species can cause infections leading to poor health and a reduction in survival [34–36]. Furthermore, early establishment of a healthy microbiome in the gastrointestinal tract (GIT) of larval fish has been reported to provide lasting health benefits via stimulation of innate immunity [2, 6, 37–39]. One of the most promising methods for establishing a healthy microbiome and preventing bacterial disease during the larval rearing stage is through the use of probiotics [36, 37, 40–42].

As newly hatched marine fish larvae have a poorly developed GIT, they must be fed on intensively cultured live prey organisms. Due to their small size, rotifers (*Brachionus plicatilis)* are typically used as the first live prey [43–45]. Rotifers are non-selective filter feeders, which make them valuable for delivering essential nutrients and probiotics to fish larvae; however, they also take up unwanted and potentially pathogenic bacteria. Rotifers are cultured at very high densities (up to 10,000 rotifers per mL of culture water) on a diet of microalgae and/or yeast [45–47], which results in high organic loads in the cultures which in turn creates an ideal environment for the rapid growth of heterotrophic bacteria which the rotifers ingest [48–50]. Common, potentially harmful bacterial genera identified in commercial rotifer cultures include *Vibrio*, *Tenacibaculum*, *Pseudomonas*, *Aeromonas* and *Photobacterium* [51–53]. Many of the *Vibrio* species are fast-growing and consequently dominate the rotifer culture. Disinfection of these opportunistic pathogenic species would allow for the subsequent targeted delivery of known exogenous probiotics, such as *Shewanella* sp., *Vibrio scophthalmi* and *Pseudoalteromonas* sp., however, previous attempts to disinfect rotifers have either been lethal to the rotifers or only minimally reduced the bacterial load [54]. Without such targeted removal, these faster growing opportunistic pathogens may inhibit any introduced probiotics due to increased competition for nutrients as well as through the production of toxins or metabolites [36, 45, 55].

Antimicrobial peptides have the potential to reduce the load of opportunistic pathogens in rotifer cultures without causing the damaging effects of antibiotics or disinfectants. This study therefore investigated if selected synthetic AMPs are effective in the brackish salt concentrations of a typical commercial rotifer culture (25‰) [47] and whether they can reduce opportunistic pathogens isolated from commercial rotifer cultures. The study also sought to determine the impacts of the AMPs on three genera of potential probiotic bacteria, *Pseudoalteromonas* sp., *Vibrio* sp. and *Shewanella* sp. that have been successfully delivered to marine fish larvae using live feeds as vectors [41, 42, 56–58].

## Methods

### Antimicrobial Peptides

The details of AMPs used in this study are shown in Table 1. All AMPs were kindly provided by the Hancock Laboratory, University of British Columbia, Canada. All AMPs supplied were manufactured by Genscript (Piscataway, New Jersey), with the exception of HHC10 and HHC36 which were manufactured by CPC Scientific (San Jose, California). All AMPs were synthesised using fluorenyl methoxy carbonyl chloride chemistry, purified to > 95% using high-pressure liquid chromatography and verified with mass spectrometry. Peptides stock solutions were prepared at a concentration of 1.3 mg mL^−1^ in deionised water and stored at −80 °C until use.

**Table 1:** Summary of the synthetic AMPs used in this study

**Peptide code**	**Sequence**	**Supplier**	**Reference**
2008	RRWIVKVRIRRR-NH_2_	Genscript	[62]
2009	KWRLLIRWRIQK	Genscript	[66]
3002	ILVRWIRWRIQW-NH_2_	Genscript	[64, 68]
3005	RRQWRGWVRIWL-NH_2_	Genscript	[68]
3008	KKWQLLIKWKLR-NH_2_	Genscript	
3011	VLQIKKVLRLLL-NH_2_	Genscript	
3018	WVGVIIKWGLKL-NH_2_	Genscript	
DJK5	vqwrairvrvir-NH_2_	Genscript	[23, 65, 68]
HHC10	KRWWKWIRW-NH_2_	CPC Scientific	[60, 63]
HHC36	KRWWKWWRR-NH_2_	CPC Scientific	[63]

### Bacterial Isolates

A range of bacterial species with the potential to be transferred to developing fish larvae as pathogens or probiotics were selected for use in the study (Table 2). Bacterial isolates were stored at −80 °C and thawed, and 25 µL of the isolates was spread onto marine salt agar (MSA) media. All isolates were incubated at 25 °C for 18 h to reach mid-log growth phase. All isolates were sub-cultured and identified using mass spectrometry (MALDI-TOF) using the BioTyper and MBT 8468 library (Bruker Pty Ltd). The isolate *Tenacibaculum discolor* was plated on Anacker and Oradal’s marine (AOM) media, incubated for 48 h and identified using the aforementioned method. Bacterial isolates were suspended in sterile saline (0.75% NaCl w/v) to 0.5 McFarland standard. Two separate bacterial solutions were made for peptides that required acetic acid and peptides that did not. From each bacterial suspension, 25 µL of this solution was added to 5 mL of Mueller Hinton broth (MHB) for testing peptides that required acetic acid, and 50 µL of the solution was added to 5 mL of MHB for peptides that did not require acetic acid; this was repeated for MHB at 25‰ and 0‰. The bacterial isolates *T. discolor* and *Pseudoalteromonas* sp. require marine salts to grow and were not added to MHB (0‰). The isolate *T. discolor* requires low nutrients to grow, therefore, a separate MHB (25‰) was prepared at a diluted ratio of 1 part MHB and 7 parts artificial seawater (25‰).

**Table 2:** Selected bacterial isolates tested against AMPs

**Bacterial isolates**	**Source**	**Isolate characteristic**	**References**
*Vibrio alginolyticus*	YTK	Pathogen	[69–71]
*Vibrio rotiferianus*	YTK gut	Pathogen	[50, 72, 73]
*Vibrio harveyi*	YTK kidney	Pathogen	[74–76]
*Tenacibaculum discolor*	Rotifer culture	Pathogen	[77, 78]
*Pseudoalteromonas* sp.	Black bream	Probiotic	[79–81]
*Shewanella* sp.	Barramundi lesions	Probiotic	[37, 82–84]
*Vibrio scophthalmi*	Black bream gut	Probiotic	[85, 86]
*Vibrio harveyi* (ATCC 35084)	ATCC	Quality control	[87, 88]
*Escherichia coli* (ATCC 25922)	ATCC	Quality control	[89, 90]

*ATCC*, American Type Culture Collection; *YTK*, yellowtail kingfish

### Minimum Inhibitory Concentration Assays

The protocol [59] was followed to determine the minimum inhibitory concentration (MIC) of the selected peptides. Two MIC assays were performed, firstly to determine whether each peptide precipitated in MHB and thereby required acetic acid adjustments (supplementary materials) and secondly, to determine the antimicrobial activity of each peptide in artificial seawater (25‰). Peptide stock solutions (1.3 mg mL^−1^) were made by diluting the peptides in sterile deionised water. MHB was made with deionised water to reach a concentration of approaching 0‰ and artificial sea salts (Sigma-Aldrich® S9883) to reach a salinity of 25‰.

### Salinity Tolerance MIC Assay

Once the acetic acid requirement for each peptide was determined (Table 3), the antimicrobial activity of each peptide was tested at a salinity of 0‰ and 25‰ for all bacterial isolates. A stock solution for peptides that required acetic acid was prepared by a 1:2 dilution in acetic acid (0.02% acetic acid w/v, containing 0.4% BSA) solution. In a 96-well polypropylene microtitre plates (Costar® 3879), 100 µL of this peptide stock was added to the first well for each bacterial isolate tested and serially diluted twofold with acetic acid (0.01% acetic acid w/v, containing 0.2% BSA). From each well, 10 µL of each peptide concentration was added to the corresponding wells of a new 96-well microtitre plate. From each of the bacterial suspensions (0‰), 90 µL was added to each well. Peptides that did not require acetic acid were diluted 1:10 in MHB, and 100 µL was added to the first well for each bacterial isolate tested. Twofold serial dilutions were made with MHB (0‰). From each of the bacterial suspensions (0‰), 50 µL was added to each well. The same method was followed for testing peptides in MHB at 25‰. All peptide concentrations ranged from 0.125 to 65 µg mL^−1^, and the bacterial suspension in each well was approximately 5 × 10^5^ CFU mL^−1^. The different salinities were used to compare any differences in antimicrobial activity in 0‰ and 25‰ water. The 96-well plates were incubated at 25 °C and read at 24 and 48 h, the MIC was visually determined using a Sensititre™ Vizion™ Digital MIC Viewing System (Thermo Scientific™), the MIC was the lowest AMP concentration that had no bacterial growth after 48 h. Each bacterium was trialled in duplicate.

**Table 3:** Summary of the MICs (µg mL^−1^) of selected AMPs against various marine bacteria with and without acetic acid

Isolates	*V. alginolyticus*		*V. rotiferianus*		*V. harveyi*		*Shewanella* sp.		*V. scophthalmi*		*V. harveyi* ATCC 35084		*E. coli* ATCC 25922	
Peptides	No acetic	Acetic	No acetic	Acetic	No acetic	Acetic	No acetic	Acetic	No acetic	Acetic	No acetic	Acetic	No acetic	Acetic
2008	1.02	0.51	1.02	0.25	1.02	1.02	0.51	0.13	0.25	0.13	1.02	1.02	4.06	2.03
2009	1.02	1.02	1.02	0.13	1.02	1.02	1.02	0.25	0.51	0.13	1.02	1.02	2.03	2.03
3002	2.03	4.06	2.03	4.06	2.03	4.06	4.06	8.13	0.51	1.02	2.03	4.06	16.25	8.13
3005	16.25	4.06	1.02	4.06	1.02	4.06	8.13	4.06	0.25	1.02	16.25	16.25	> 65	> 65
3008	2.03	1.02	1.02	2.03	4.06	4.06	16.25	8.13	1.02	0.51	16.25	16.25	> 65	> 65
3011	4.06	4.06	4.06	4.06	32.5	16.25	16.25	16.25	2.03	4.06	8.13	16.25	8.13	16.25
3018	4.06	8.13	4.06	8.13	16.25	16.25	8.13	16.25	2.03	0.25	8.13	16.25	16.25	> 65
DJK5	4.06	4.06	1.02	1.02	4.06	2.03	1.02	1.02	0.51	0.51	2.03	4.06	8.13	4.06

### Rotifer Trial

To determine whether antimicrobial peptides were effective at reducing bacterial density load in a biological environment, commercial rotifer culture water was treated with the two AMPs from the MIC assays under high salt condition, peptides 2009 and 3002. These were the only peptides that reduced bacterial growth of pathogens *V. rotiferianus* and *T. discolor*, respectively. The antimicrobial peptides were inoculated into the rotifer culture water at the maximum MIC value of 65 µg mL^−1^ individually and in combination.

Briefly, a 12-well polypropylene pipette reservoir (Z370843-8EA, Scienceware®) was used to test the selected peptides and rotifer culture. In each well, the peptide stock (1.3 mg mL^−1^) was diluted with the rotifer culture water to a final concentration of 65 µg mL^−1^. For each treatment, there was a corresponding control which excluded the peptide. Where the peptides were trialled in combination, each well included both peptides to a final peptide concentration of 65 µg mL^−1^. All concentrations were tested in triplicate.

The rotifer culture water was sourced from a commercial stock (ca. 5.59 ± 0.02 Log CFU mL^−1^), the rotifers were harvested by pouring 50 mL of rotifer culture through an 80-µm sieve. The rotifer culture water was collected and measured into the reservoir wells, this was a total volume of 3 mL. The peptides were diluted into each corresponding well, and samples were taken hourly for 3 h. At each hour, 100 µL of rotifer culture water was removed from the corresponding reservoir wells and serially diluted tenfold in sterilised seawater (25‰) down to 1 × 10^−3^ and plated on marine agar. Plates were incubated at 25 °C for 24–48 h. A total bacterial count was recorded for each hour for all the treatments.

### Statistical Analysis

The effect of the AMPs on final bacteria density after culture was analysed by two-way ANOVA. Data were log-transformed and analysed using JMP Software (SAS, Version 16).

## Results

### MIC assays under low salt conditions

Peptides 3008, 3018, 2008 and 2009 required addition of acetic acid whereas 3005, 3011, 3002 and DJK5 did not (Table 3). The AMPs 2008, 2009, 3002, HHC10 and DJK5 had an MIC value between 1 and 10 µg mL^−1^ for all or most of the bacterial isolates in low salt conditions (Table 3). The MICs for the remaining peptides ranged from 0.25 to greater than 65 µg mL^−1^, and the majority of these lower MICs were all attributed to the most susceptible bacterium, *Vibrio scophthalmi*. The peptides 2008 and 2009 were the most effective against the bacterial pathogens *Vibrio alginolyticus* (0.51 and 1.02 µg mL^−1^), *V. rotiferianus* (0.25 and 0.13 µg mL^−1^) and *V. harveyi* (1.02 µg mL^−1^), respectively. However, the probiotic strains were more susceptible to these peptides (0.13–0.25 µg mL^−1^).

### MIC Assays Under High Salt Conditions

The use of 25‰ artificial seawater hindered the ability of the AMPs to inhibit the bacterial growth for all the strains tested (Table 4). The majority of the peptides had an MIC of greater than 65 µg mL^−1^, which was the maximum value in the MIC assay. The only exceptions were 3008 (32.5 µg mL^−1^), 2009 (32.5 µg mL^−1^) and 3002 (32.5 µg mL^−1^). Of these peptides, only 2009 and 3002 were able to inhibit the growth of pathogenic isolates, *V. rotiferianus* and *T. discolor*, respectively.

**Table 4:** Summary of the MICs (µg mL^−1^) of selected AMPs against various marine bacteria in low and high salt concentrations

Isolates	*V. alginolyticus*		*V. rotiferianus*			*V. harveyi*		*Shewanella* sp.		*V. scophthalmi*		*V. harveyi* ATCC 35084		*E. coli* ATCC 25922		*T. discolor*	*Pseudoalteromonas* sp.
Peptides / Salinity (‰)	0	25		0	25	0	25	0	25	0	25	0	25	0	25	25	25
2008	0.13	65		0.25	65	1.02	> 65	0.51	65	0.25	65	1.02	> 65	4.06	> 65	> 65	65
2009	1.02	65		0.13	32.5	1.02	> 65	0.51	> 65	< 0.13	32.5	1.02	> 65	2.03	> 65	> 65	> 65
3002	8.13	> 65		8.13	> 65	8.13	> 65	8.13	> 65	2.03	> 65	8.13	> 65	65	> 65	32.5	65
3005	2.03	> 65		1.02	> 65	4.06	> 65	8.13	65	0.13	65	4.06	> 65	4.06	> 65	> 65	> 65
3008	2.03	> 65		2.03	> 65	4.06	> 65	16.25	32.5	< 0.13	32.5	8.13	> 65	8.13	> 65	> 65	> 65
3011	4.06	> 65		4.06	> 65	8.13	> 65	8.13	> 65	1.02	> 65	8.13	> 65	16.25	> 65	> 65	> 65
3018	2.03	> 65		8.13	> 65	16.25	> 65	16.25	> 65	1.02	> 65	32.5	> 65	65	> 65	> 65	> 65
DJK5	4.06	> 65		1.02	> 65	4.06	> 65	2.03	> 65	0.51	> 65	2.03	> 65	2.03	> 65	> 65	> 65
HHC10	2.03	> 65		2.03	> 65	8.13	> 65	4.06	> 65	2.03	> 65	4.06	> 65	4.06	> 65	> 65	> 65
HHC36	> 65	> 65		> 65	> 65	32.5	> 65	1.01	> 65	2.03	> 65	32.5	> 65	2.03	> 65	> 65	> 65

### Rotifer Trials

**Fig. 1:**
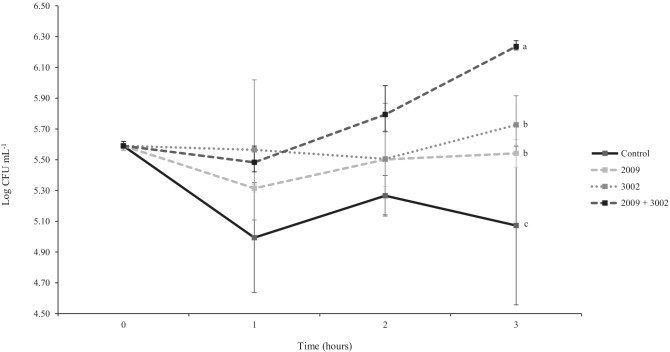
The mean total bacterial count (Log CFU mL^−1^ ± S.D.) of commercial rotifer culture water treated with two antimicrobial peptides (2009 and 3002), individually or combined at 65 µg mL^−1^ compared to a control culture with no peptides over 3 h. Letters signify significant difference at the end of the experiment

The initial total bacterial concentration of rotifer culture water was 5.59 ± 0.02 LogCFU mL^−1^. A significant increase in bacterial concentration occurred over 3 h, with the peptide-treated cultures showing more growth (*P* = 0.03; Fig. 1). All treatments were higher than the control treatment (LSM = 5.11 LogCFU mL^−1^), and this was the only culture to decline in bacterial growth. There was a significant effect of treatment on the bacterial concentration (*P* < 0.001), with the AMP combination treatment having the least impact on the bacterial load (LSM = 5.84 LogCFU mL^−1^). There was no significant difference between AMPs 3002 (LSM = 5.60 LogCFU mL^−1^) and 2009 (LSM = 5.45 LogCFU mL^−1^) on the overall bacterial load after 3 h.

## Discussion

This study investigated the antibacterial susceptibility of synthetic AMPs against putative pathogenic and probiotic bacteria found within rotifer cultures. The assays were conducted at a salinity of 25‰ to determine if these peptides had any antimicrobial effect at the salt concentration used in commercial rotifer cultures. The use of synthetic AMPs could be a novel technique to aid the bioencapsulation of live feeds with probiotic bacteria. The major findings of this study were high salt concentrations decreased the MIC value of the selected AMPs against common marine bacteria found in rotifer cultures; the majority of the selected AMPs had an MIC between 1 and 10 µg mL^−1^ in low salt concentrations, and the peptides 2009 and 3002 were ineffective at reducing the number of bacteria in rotifer culture water.

This study determined that high salt concentrations negatively affected the antimicrobial activity of the selected peptides. All the peptides had an increased MIC when tested at a high salt concentration (25‰) compared to a low salt concentration. These results agree with other studies that identified high salt concentrations negatively impacted the antimicrobial activity of synthetic AMPs [27, 31, 60, 61]. The presence of some cations in solutions significantly diminished the peptides’ antimicrobial ability [18, 19, 60]. In seawater, molecules such as sodium, calcium and magnesium can cause an AMP to be ineffective, because the increased salt concentration tends to reduce the peptide’s potency [19, 62]. For example, Cherkasov et al. [63] found the peptide HHC10 was extremely potent against antibiotic resistant microbes (i.e. methicillin-resistant *Staphylococcus aureus* (MRSA) and vancomycin-resistant enterococci), with MIC values of 0.3–11 µM in MHB. This same peptide was potent against the fish pathogens *Aeromonas hydrophilia* and *Yersinia ruckeri*, in freshwater, with low MIC values of 10–20 µM, however, when the authors included half strength seawater (300 mM NaCl), the MIC value increased to > 80 µM [60]. In this study, HHC10 had an MIC range of 2.03–8.13 µg mL^−1^ at a low salt concentration, which was a similar range to those reported by Cherkasov et al. [63]. When artificial seawater (25‰) was added to the growth medium, the MIC value increased to > 65 µg mL^−1^, suggesting that salts negatively affected the antimicrobial activity of the peptide. In relation to the other peptides, HHC36 had a similar potency as HHC10 in [63], but in this study, the HHC36 was not as active as HHC10 at low salt concentrations against the pathogenic *Vibrio* species. Although the selected peptides were ineffective at high salt concentrations, in low salt concentrations, the majority of the peptides had MIC values that ranged between 0.13 and 16.25 µg mL^−1^, with peptides 2008, 2009 and DJK5 having MIC values as low as 2.06–4.06 µg mL^−1^. It was clear that the high salt concentration caused a loss of antimicrobial activity in all peptides tested in this study. Multiple studies have previously investigated modifying AMPs to have a higher salt tolerance, however, this tolerance was generally increased to a physiological salt concentration (100–300 mM NaCl) because the majority of antimicrobial peptide research is focused on human disease prevention. Therefore, this salt tolerance is very low compared to the salinities in marine aquaculture (i.e. 450–600 mM NaCl).

The peptides 2009 and 3002 were tested in a biological setting and were ineffective at reducing the total bacterial load present in rotifer culture water, instead increasing the total number of bacteria. The MIC assay under high salt concentration identified these peptides as being effective against *V. rotiferianus* and *T. discolor* at a high peptide concentration (65 µg mL^−1^), however, the two bacterial species were not identified in the control rotifer culture, and the reduction of their presence cannot be quantified. In relation to other AMP research, the majority of AMPs are currently tested in vitro and therefore in vivo studies are limited, especially in marine organisms. Of all the peptides tested in this study, only the peptides DJK5 and 3002 were previously studied in terrestrial organisms. In both peptides, active antibiofilm characteristics were identified from laboratory trials and successfully reduced MRSA and *P. aeruginosa* bacterial infections in mice and nematodes, respectively [23, 64, 65]. Although peptide 3002 was effective at reducing bacterial infections in terrestrial organisms, this study reported the peptide was unsuccessful at reducing bacteria numbers in rotifer cultures.

A study by Haney [66] determined the aggregation properties of the innate defence regulator (IDR) peptides 1018, 1002 and HH2 in different media such as salts and serum. These IDR peptides are synthetic analogues to natural host defence peptides (HDPs) found in organisms. The study reported that these peptides exhibit immunomodulatory functions and antibiofilm potency but also have the tendency to aggregate in the presence of serum and high salt concentrations, causing the peptides to be ineffective. Second-generation peptides are synthetically rearranged IDRs that are created to combat unwanted traits such as aggregation. Multiple derivatives including peptides 2008 [62] and 2009 [66] of the aforementioned model IDR peptides were made to understand if the sequence was responsible for the hydrophobicity that causes aggregation. Both peptides caused cytotoxicity, however, only peptide 2008 retained the immunological properties and effectively reduced MRSA biofilm.

Limitations of this study included the exclusion of rotifers in the culture water and harmful traits such as cytotoxicity not being reported on, this aspect should be considered in future research because traits such as cytotoxicity limit the application of peptides as a therapeutic. Based on these findings, the use of AMPs in rotifer cultures caused the number of bacteria in the rotifer culture to increase, rather than decrease. Synthetic peptide modifications have improved the potential application of peptides as an antimicrobial agent, however, there are issues surrounding the sensitivity of these synthetic analogues to salts, which have been improved to tolerate physiological salt concentrations. For example, Kerenga et al. [67] found that the synthetic peptide ZmD32 had a high salt tolerance (100 mM NaCl), but when magnesium chloride (5 mM) was introduced, the activity of the peptide decreased sevenfold. Similar results were recorded by Friedrich et al. [25] for the peptides CP26, CP29 and CEME which were active against *E. coli* and *P. aeruginosa* at higher salt concentrations (100–300 mM NaCl), however, when divalent cations (MgCl_2_) were introduced, the antimicrobial activity was reduced up to eightfold. Incorporating ions rather than sea salt in the MIC assays used in this study would reveal what ions may be inhibiting the peptides. A synthetic peptide was designed based on a defence protein found in sea squirts (*Ciona intestinalis*), it was found that peptide Ci-MAM-A24 had antimicrobial activity against human and marine pathogens up to 450 mM NaCl [24]. The presence of divalent cations in seawater has severely limited peptides’ use in marine environments and is an aspect of peptide modifications that requires improvement. Screening synthetic peptides designed from natural peptides located in marine organisms may further improve the tolerance to high salt and potentially divalent cations in seawater. The application of AMPs in aquaculture is limited to low salinity environments. Further research on synthetic peptide modification is required for AMPs to be used in salinities relevant to marine aquaculture systems.

This study determined that the selected synthetic AMPs were not effective in brackish salt concentrations of a typical commercial rotifer culture (25‰). The peptides 2009 and 3002 were selected to be tested in rotifer cultures, however, they did not reduce the opportunistic pathogens detected in commercial rotifer cultures. The majority of the AMPs had a more effective antimicrobial effect on the three species of probiotic bacteria, *Pseudoalteromonas*, *Vibrio* and *Shewanella* in low and high salt concentrations. Overall, antimicrobial peptide research is in early development, and understanding their prophylactic potential in aquatic organisms is not well understood.

## Supplementary Information

Below is the link to the electronic supplementary material.

Supplementary file1 (DOCX 13 KB)

## Data Availability

The datasets generated during and/or analysed during the current study are available from the corresponding author on reasonable request.
