# Supplementary material for: Assessing the Activity of Antimicrobial Peptides Against Common Marine Bacteria Located in Rotifer (Brachionus plicatilis) Cultures
Source: Probiotics Antimicrob Proteins. 2022 May 25;14(4):620–9. doi: 10.1007/s12602-022-09928-2 (PMC9246773; doi:10.1007/s12602-022-09928-2)
Supplement: Supplementary file 1 — Supplementary file1 (DOCX 13 KB) [file 12602_2022_9928_MOESM1_ESM.docx]

**Supplementary material**

***Acetic acid solubility adjustments***

A broth microdilution protocol [59] was followed to test whether the AMPs precipitated in MHB. The isolates *T. discolor* and *Pseudoalteromonas* were excluded from this assay because they required marine salts for growth. The peptides HHC10 and HHC36 previously required acetic acid adjustments according to [63] and were not trialled in this assay. To test if peptides required acetic acid, each peptide was diluted 1:2 in acetic acid (0.02% acetic acid w/v, containing 0.4% BSA) solution. In a 96-well polypropylene microtitre plates (Costar^®^ 3879), 100 µL of this peptide stock was added to the first well for each bacterial isolate tested and serially diluted 2-fold with acetic acid (0.01% acetic acid w/v, containing 0.2% BSA). From each well, 10 µL of each peptide concentration was added to the corresponding wells of a new 96-well microtitre plate. From each of the bacterial suspensions in MHB (0‰), 90 µL was added to each well. To test if peptides did not require acetic acid, each peptide was diluted 1:10 in MHB, 100 µL was added to the first well and two-fold serial dilutions were made with MHB (0‰). From each of the bacterial suspensions in MHB (0‰), 50 µL was added to each well. All peptide concentrations ranged from 0.125 - 65µg mL^-1^ and the bacterial suspension in each well was approximately 5 × 10^5^ CFU mL^-1^. Antimicrobial peptides that have an MIC value two or more dilutions higher in the assay with no acetic acid, compared to the assay with acetic acid, require acetic acid adjustments [59]. The 96-well plates were incubated at 25⁰C and read at 24 and 48 hours. The MIC was visually determined using a Sensititre™ Vizion™ Digital MIC Viewing System (Thermo Scientific™), and the lowest AMP concentration that had no bacterial growth was recorded as the MIC after 48 hours. Each bacterium was trialled in duplicate.
